# The Prophage and Us—Shiga Toxin Phages Revisited

**DOI:** 10.3390/pathogens12020232

**Published:** 2023-02-02

**Authors:** Herbert Schmidt, Maite Muniesa

**Affiliations:** 1 Department of Food Microbiology, Institute of Food Science and Biotechnology, University of Hohenheim, 70599 Stuttgart, Germany; 2 Departament de Genètica, Microbiologia i Estadística, Universitat de Barcelona, 08028 Barcelona, Spain

The authors first met in 1998 at the University of Würzburg, Germany, at the Institute of Hygiene and Microbiology, in Helge Karch’s lab, where Herbert Schmidt worked as a PostDoc and Maite Muniesa visited the lab for a postdoctoral research stay to work on phages encoding Shiga toxin 2e (Stx2e) [1]. Since that time, we have been more or less, as much as our university duties allow, connected by Stx-phage research. 

Initially described in the early 1980s, Shiga toxin-converting bacteriophages (Stx-phages) have been the subject of numerous publications [2,3]. The ability to produce Stx, the major pathogenicity factor of enterohemorrhagic *E. coli* (EHEC), seems to be essentially connected to the location of the Shiga toxin genes (*stx*) in the genome of lysogenic phages, found always in a similar location within the late transcribed region, and upstream of the lysis and capsid genes [4,5,6,7,8,9,10,11]. Stx-phages are double-stranded DNA tailed phages showing a lambdoid or a non-lambdoid genome structure. To the best of our knowledge, *stx* genes have never been found in other mobile genetic elements other than phages, such as plasmids or pathogenicity islands [12,13,14,15,16]. Although the genetics and function of Stx-phages have been described in many publications, some basic questions remain still open, for example, (1) why are Stx-phages so successful in terms of evolution and spread among *E. coli* strains? (2) why do Stx-phages occur mainly in enteropathogenic *E. coli* strains but not, or only accidentally, in others such as extraintestinal pathogenic *E. coli* (EXPEC), (3) which advantage do EHEC strains have from the lysogenic state carrying single or multiple Stx-phages?

As temperate phages, Stx-phages occupy distinct integration sites in the bacterial host genomes [4]. EHEC bacteria may carry one single or multiple Stx-phages; as far as we know, up to 21 prophages have been counted, and sometimes they are integrated in already resident phages. The lengths and genetic composition of these phages differ enormously, and many are fully functional prophages; moreover, cryptic prophages which have lost parts of their genes can be found [15]. The data of Khan et al. [17] suggested that in incomplete prophages, mostly the genes for the replication, packaging, and release of phage particles have been lost, without them being unable to induce and form functional phage particles. The lysogenic state seems so persistently maintained that it suggests that this association should be advantageous for both the host and the phage [17].

The Stx family consists of two larger groups, Stx1, which is almost identical to Stx of *Shigella*, and Stx2, with numerous variants [18]. The variants have been successfully spread among *E. coli* strains, where all known *stx* variants are found, always encoded in the genome of complete or defective prophages [19,20]. In contrast, *stx* variants are only randomly found in *Shigella* and in close relatives such as *Serratia marcescens* and *Citrobacter freundii*. The enormous abundance of *stx* genes in prophages in *E. coli* strains, which are present in many animals, raises the question of the advantage of *stx* particularly for *E. coli*. 

In contrast, Stx-prophages were described in *Shigella sonnei*, but it was initially thought that the *stx* operon was mainly chromosomally encoded in *Shigella dysenteriae* [21]. It is now clear that in all cases, the *stx* genes are also surrounded by prophage sequences, even if these are complete or remnant [22]. While few Stx-prophages are inducible, many Stx-phages have become defective particularly in *Shigella* spp, [23], leading to the initial incorrect assumption that *stx* was chromosomally encoded in this genus. Unlike *E. coli*, it was proposed that all Stx-producing *S. dysenteriae* 1 derive from a clone that resulted from a single phage-integration event [23]. This original Stx-encoding lambdoid prophage subsequently became defective because of a loss of phage sequences following multiple IS element insertions and IS-mediated rearrangements, which commonly occurs in *Shigella* [24], and when located close to the *stx* operon [22], even resulting in the loss of *stx*. 

Taken together, many *Shigella* strains seem to have received and then lost the *stx* genes in their evolutionary past. The loss of *stx* provided advantages to the bacteria for better adaptation to the human hosts, since being a more severe pathogen is not the best strategy for the organism’s long-term survival. Alternatively, the integration, retention, and expression of certain new introduced virulence genes might depend on their interaction between them and the bacterial genomic background [25]. In other hosts, as is the case with intestinal *E. coli* strains, besides the acquisition of a powerful virulence determinant such as Stx, the presence of Stx-phages seems to enhance the colonization and survival of strains residing in certain environments [26]. In addition to these, other advantages can be envisaged [4], the extent of which is not yet fully elucidated [27]. Perhaps the right genomic background to stably retain and express Stx occurs preferentially in enteropathogenic *E. coli*, in *S. dysenteriae* type 1, and less frequently in some non-*S. dysenteriae* type 1 strains [28]. In contrast, Stx-phages rarely encounter the right background in EXPEC, or in many other *Shigella* species, despite the fact that some of these are susceptible to being lysogenized by Stx-phages under laboratory conditions [29,30].

In a multitude of STEC serotypes, it is widely known that not only one, but multiple prophages, including more than one Stx-phage, can co-exist [19]. These multiple Stx-phages show a diversity of lysis-lysogeny regulatory systems, but all have in common a conserved lytic system always adjacent to *stx* genes [20], which guarantees the *stx* expression during prophage excision. 

The lack of phage immunity that suppresses the prevention of superinfection [31], the use of diverse integration sites for different Stx-phages [32], the integration in tandem of two Stx-phages sharing the same *attB* site [15], and even the recently revealed “prophage-in-prophage” ability, in which a Stx-phage integrates within the *attB* region present in other prophages, might be the main causes explaining the presence of more than one Stx-phage in a single cell [15]. The pros and cons of carrying more than one Stx-phage are still under discussion, and in the presence of two or more Stx-prophages, some authors propose an enhanced Stx production [16], while others show a down-regulated *stx* expression [33,34]. In fact, both options might be beneficial for the bacteria. In the case of a higher Stx production, the acceptance of more than one Stx-prophage allows the strain to become more pathogenic. In the case of down-regulated Stx production, the presence of multiple Stx-phages could allow a better survival and a milder pathogenicity that, as discussed above, seems a plausible long-term survival strategy. It is possible that both options can occur, depending on the phage induction levels, the host genetic background and its protein expression capacity, and the environmental factors of the host strain reservoir. It remains unclear, however, if benefits can be derived for the phages co-existing within the same host, other than allowing recombination events between phages that ensure the genetic variability and evolvability of the Stx-phages [20].

Exploring the origin of *stx* before the original toxin described in *S. dysenteriae* type 1, Shiga toxins belong to the enzyme group of ribosome-inactivating proteins (RiP) (E.C. 3.2.2.22) and are classified as type 2 RiPs with two chains, consisting of a single enzymatic subunit, connected to five receptor-binding B-subunits. Single-chain type 1 RiPs occur frequently in plants and show antiviral, antifungal, and insecticidal activity [35,36]. Interestingly, the Stx A-subunit shows some homology to one of these RiPs, ricin, and when comparing Stx A-subunit with ricin, both show a similar molecular weight, similar crystal structure, and highly conserved amino acids involved in catalysis [37,38]. These similarities initially pointed towards RiPs as a possible origin of Stx. However, experimental evidence [39] showed that ricin, Stx1, and Stx2 have completely different B subunits and interact differently with ribosomes [40,41,42]. Moreover, other similar proteins, such as abrin, modeccin, volkensin, or viscumin, are examples of RiPs that use the same method of entry and mechanism of action and that, far from having a common ancestor, have probably evolved via convergent evolution [43], a separate evolution resulting in analogous structures. This hypothesis discards a possible relationship between Stx and RiPs, suggesting only that, in nature, organisms independently find the same solutions to solve similar problems.

Research that focuses on the molecular biology, function, and impact of Shiga toxin phages is fascinating and worth continuing. The ecological potential and pathogenicity of EHEC strains resides, besides chromosomal genes, on the function of mobile genetic elements, and among these, Stx-encoding phages, which are not yet well understood. Multi-faceted interdisciplinary research is needed to pave the way for a deeper understanding of this important group of genetic elements, and these old entities appear today as highly topical as ever. The increase in hybrid EHEC/enteroaggregative *E. coli* strains carrying Stx-phages and expressing Stx and the outbreaks caused by such strains underlines this statement. In this editorial, we intended to open the discussion about these yet unanswered questions, with the hope to highlight the enormous impact of Stx-phages and provide a primer for further Stx-phage research.

## References

[1] Muniesa M., Recktenwald J., Bielaszewska M., Karch H., Schmidt H. (2000). Characterization of a Shiga toxin 2e-converting bacteriophage from an *Escherichia coli* strain of human origin. Infect. Immun..

[2] O’Brien A.D., Laveck G.D., Thompson M.R., Formal S.B. (1982). Production of *Shigella dysenteriae* type 1-like cytotoxin by *Escherichia coli*. J. Infect. Dis..

[3] O’Brien A.D., Newland J.W., Miller S.F., Holmes R.K., Smith H.W., Formal S.B. (1984). Shiga-like toxin-converting phages from *Escherichia coli* strains that cause hemorrhagic colitis or infantile diarrhea. Science.

[4] Rodríguez-Rubio L., Haarmann N., Schwidder M., Muniesa M., Schmidt H. (2021). Bacteriophages of Shiga toxin-producing *Escherichia coli* and their contribution to pathogenicity. Pathogens.

[5] Herold S., Karch H., Schmidt H. (2004). Shiga toxin-encoding bacteriophages—Genomes in motion. Int. J. Med. Microbiol..

[6] Allison H.E. (2007). Stx-Phages: Drivers and mediators of the evolution of STEC and STEC-like pathogens. Fut. Med..

[7] Yokoyama K., Makino K., Kubota Y., Watanabe M., Kimura S., Yutsudo C.H., Kurokawa K., Ishii K., Hattori M., Tatsuno I. (2000). Complete nucleotide sequence of the prophage VT1-Sakai carrying the Shiga toxin 1 genes of the enterohemorrhagic *Escherichia coli* O157:H7 strain derived from the Sakai outbreak. Gene.

[8] Karch H., Schmidt H., Janetzki-Mittmann C., Scheef J., Kröger M. (1999). Shiga toxins even when different are encoded at identical positions in the genomes of related temperate bacteriophages. Mol. Gen. Genet..

[9] Plunkett G., Rose D.J., Durfee T.J., Blattner F.R. (1999). Sequence of Shiga toxin 2 phage 933W from *Escherichia coli* O157:H7: Shiga toxin as a phage late-gene product. J. Bacteriol..

[10] Neely M.N., Friedman D.I. (1998). Functional and genetic analysis of regulatory regions of Coliphage H-19B: Location of Shiga-like toxin and lysis genes suggest a role for phage functions in toxin release. Mol. Microbiol..

[11] Neely M.N., Friedman D.I. (1998). Arrangement and Functional identification of genes in the regulatory region of lambdoid phage H-19B, a carrier of a Shiga-like toxin. Gene.

[12] Kyle J.L., Cummings C.A., Parker C.T., Quiñones B., Vatta P., Newton E., Huynh S., Swimley M., Degoricija L., Barker M. (2012). *Escherichia coli* serotype O55:H7 diversity supports parallel acquisition of bacteriophage at Shiga toxin phage insertion sites during evolution of the O157:H7 lineage. J. Bacteriol..

[13] Hayashi T., Makino K., Ohnishi M., Kurokawa K., Ishii K., Yokoyama K., Han C.G., Ohtsubo E., Nakayama K., Murata T. (2001). Complete genome sequence of enterohemorrhagic *Escherichia coli* O157:H7 and genomic comparison with a laboratory strain K-12. DNA Res..

[14] Lorenz S.C., Gonzalez-Escalona N., Kotewicz M.L., Fischer M., Kase J.A. (2017). Genome sequencing and comparative genomics of enterohemorrhagic *Escherichia coli* O145:H25 and O145:H28 reveal distinct evolutionary paths and marked variations in traits associated with virulence & colonization. BMC Microbiol..

[15] Nakamura K., Ogura Y., Gotoh Y., Tetsuya H. (2021). Prophages integrating into prophages: A mechanism to accumulate type III secretion effector genes and duplicate Shiga toxin-encoding prophages in *Escherichia coli*. PLoS Pathog..

[16] Fogg P.C.M., Saunders J.R., Mccarthy A.J., Allison H.E. (2012). Cumulative effect of prophage burden on Shiga toxin production in *Escherichia coli*. Microbiology.

[17] Khan A., Burmeister A.R., Wahl L.M. (2020). Evolution along the parasitism-mutualism continuum determines the genetic repertoire of prophages. PLoS Comput. Biol..

[18] Scheutz F., Teel L.D., Beutin L., Piérard D., Buvens G., Karch H., Mellmann A., Caprioli A., Tozzoli R., Morabito S. (2012). Multicenter evaluation of a sequence-based protocol for subtyping Shiga toxins and standardizing Stx nomenclature. J. Clin. Microbiol..

[19] Shaaban S., Cowley L.A., McAteer S.P., Jenkins C., Dallman T.J., Bono J.L., Gally D.L. (2016). Evolution of a zoonotic pathogen: Investigating prophage diversity in enterohaemorrhagic *Escherichia coli* O157 by Long-read sequencing. Microb. Genom..

[20] Pinto G., Sampaio M., Dias O., Almeida C., Azeredo J., Oliveira H. (2021). Insights into the genome aarchitecture and evolution of Shiga toxin encoding bacteriophages of *Escherichia coli*. BMC Genom..

[21] Fogolari M., Mavian C., Angeletti S., Salemi M., Lampel K.A., Maurelli A.T. (2018). Distribution and characterization of Shiga toxin converting temperate phages carried by *Shigella flexneri* in Hispaniola. Infect. Genet. Evol..

[22] Unkmeir A., Schmidt H. (2000). Structural analysis of phage-borne stx genes and their flanking sequences in Shiga toxin-producing *Escherichia coli* and *Shigella dysenteriae* type 1 strains. Infect. Immun..

[23] Greco K.M., McDonough M.A., Butterton J.R. (2004). Variation in the Shiga toxin region of 20th-century epidemic and endemic *Shigella dysenteriae* 1 strains. J. Infect. Dis..

[24] Yang F., Yang J., Zhang X., Chen L., Jiang Y., Yan Y., Tang X., Wang J., Xiong Z., Dong J. (2005). Genome dynamics and dversity of *Shigella* species, the etiologic agents of bacillary dysentery. Nucleic Acids Res..

[25] Escobar-Páramo P., Clermont O., Blanc-Potard A.B., Bui H., le Bouguénec C., Denamur E. (2004). A specific genetic background Is required for acquisition and expression of virulence factors in *Escherichia coli*. Mol. Biol. Evol..

[26] Saile N., Schwarz L., Eißenberger K., Klumpp J., Fricke F.W., Schmidt H. (2018). Growth Advantage of *Escherichia coli* O104:H4 strains on 5-*N*-Acetyl-9-*O*-Acetyl neuraminic acid as a carbon source Is dependent on heterogeneous phage-borne NanS-p esterases. Int. J. Med. Microbiol..

[27] Zuppi M., Tozzoli R., Chiani P., Quiros P., Martinez-Velazquez A., Michelacci V., Muniesa M., Morabito S. (2020). Investigation on the evolution of Shiga Toxin-converting phages based on whole genome sequencing. Front. Microbiol..

[28] Zhi S., Parsons B.D., Szelewicki J., Yuen Y.T.K., Fach P., Delannoy S., Li V., Ferrato C., Freedman S.B., Lee B.E. (2021). Identification of Shiga-toxin-producing *Shigella* infections in travel and non-travel related cases in Alberta, Canada. Toxins.

[29] Tozzoli R., Grande L., Michelacci V., Ranieri P., Maugliani A., Caprioli A., Morabito S. (2014). Shiga toxin-converting phages and the emergence of new pathogenic *Escherichia coli*: A world in motion. Front. Cell Infect. Microbiol..

[30] Gray M.D., Lacher D.W., Leonard S.R., Abbott J., Zhao S., Lampel K.A., Prothery E., Gouali M., Weill F.X., Maurelli A.T. (2015). Prevalence of Stx-producing *Shigella* species isolated from french travelers returning from the caribbean: An emerging pathogen with international implications. Clin. Microbiol. Infect..

[31] Serra-Moreno R., Jofre J., Muniesa M. (2007). Insertion site occupancy by Stx2 bacteriophages depends on the locus availability of the host strain chromosome. J. Bacteriol..

[32] Henderson S.T., Singh P., Knupp D., Lacher D.W., Abu-Ali G.S., Rudrik J.T., Manning S.D. (2021). Variability in the occupancy of *Escherichia coli* O157 integration sites by Shiga toxin-encoding prophages. Toxins.

[33] Serra-Moreno R., Jofre J., Muniesa M. (2008). The CI repressors of Shiga toxin-converting prophages are involved in coinfection of *Escherichia coli* strains, which causes a down regulation in the production of Shiga toxin 2. J. Bacteriol..

[34] Ogura Y., Mondal S.I., Islam M.R., Mako T., Arisawa K., Katsura K., Ooka T., Gotoh Y., Murase K., Ohnishi M. (2015). The Shiga toxin 2 production level in enterohemorrhagic *Escherichia coli* O157:H7 Is correlated with the subtypes of toxin-encoding phage. Sci. Rep..

[35] Jiménez P., Tejero J., Cordoba-Diaz D., Quinto E.J., Garrosa M., Gayoso M.J., Girbés T. (2015). Ebulin from Dwarf Elder (*Sambucus Ebulus* L.): A Mini-Review. Toxins.

[36] Stirpe F. (2004). Ribosome-inactivating proteins. Toxicon.

[37] Fraser M.E., Chernaia M.M., Kozlov Y.v., James M.N.G. (1994). Crystal structure of the holotoxin from *Shigella dysenteriae* at 2.5 Å resolution. Nat. Struct. Biol..

[38] Katzin B.J., Collins E.J., Robertus J.D. (1991). Structure of ricin A-chain at 2.5 Å. Proteins Struct. Funct. Bioinf..

[39] Tumer N.E., Li X.P. (2012). Interaction of Ricin and Shiga toxins with ribosomes. Curr. Top. Microbiol. Immunol..

[40] Chiou J.C., Li X.P., Remacha M., Ballesta J.P.G., Tumer N.E. (2008). The ribosomal stalk Iis required for ribosome binding, depurination of the rRNA and cytotoxicity of ricin A chain in *Saccharomyces cerevisiae*. Mol. Microbiol..

[41] Chan Y.S., Ng T.B. (2016). Shiga Toxins: From structure and mechanism to applications. Appl. Microbiol. Biotechnol..

[42] Chiou J.C., Li X.P., Remacha M., Ballesta J.P.G., Tumer N.E. (2011). Shiga toxin 1 is more dependent on the P proteins of the ribosomal stalk for depurination activity than Shiga toxin 2. Int. J. Biochem. Cell Biol..

[43] Lapadula W.J., Sanchez-Puerta M.V., Juri Ayub M. (2012). Convergent evolution led ribosome inactivating proteins to interact with ribosomal stalk. Toxicon.

